# Supplementary material for: The role of iron deficiency and factors associated with anemia during pregnancy in Southeastern Tigray, Ethiopia, 2020
Source: PLoS One. 2025 Feb 10;20(2):e0318275. doi: 10.1371/journal.pone.0318275 (PMC11809803; doi:10.1371/journal.pone.0318275)
Supplement: Attachment — Submitted filename: plose review report february 2024.docx [file pone.0318275.s002.docx]

Thanks for inviting me to review article entitled “**Contribution of iron deficiency to anemia and Factors associated with anemia during pregnancy in Southeastern Tigray, Ethiopia, 2020”**

I read the paper with interest and it was really good, however there are area need to improve and required further explanation.

**Introduction part**

Some of your data in the introduction part was old enough kindly let you replace it with the new information. The claim that contribution of anemia is unknown looks like exaggerated. we know many study conducted in Ethiopia and in Tigray region too.

**Methods.**

a. The study setting for this particular study was not clear? Your study design was facility based cross sectional study however in which particular setting that the study conducted was not noted
b. Your study population need to be well specific. It stated “pregnant women “ …..it was better if you clearly specified those pregnant women and from where you recruited them? Do you mean pregnant women come for ANC? Or pregnant women come hospital for other purpose? It recommended if you well specified your study population separately.
c. Like that of study population, your study failed to specified the exact inclusion criteria.
d. Sample size determination you used was from the EDHS 2016 which was population based survey, why you didn’t used the recent study with similar study population
e. The calculated sample size was 317. and why not considered the non-response rate. it is common to use include about 10 %or 5% the sample size as non-response rate.
f. The sampling procedure was not clear. Need to specified each. Put number for each steps.
g. Your study variable is contribution of anemia or anemia???
h. The tools for data collection should be cited eg DDS
i. How you check the validity of Hem cue measurement. what was its principle. please specify amount of blood you collected by cuvette \
j. It stated *“Finally the punctured part was wiped for any remaining blood from the third drop and covered with an alcohol swab.”* **Do you think that was simple acceptable you covered that punctured site with alcohol swap??**
k. How you measure adherence to IFA and the knowledge was not clear
l. What you did for those anemic pregnant women

**Result**

1. “Number of ANC Visit” the word visit should replaced by contact
2. In table to of the contraceptive it says “Jagule” I never encounter such named contraceptive kindly specify it
3. It recommended that that px women should take meal about four time in day however in your case about three-fourth of participants get food less than three time. Could you please justify it?
4. Only about 28% of your participant had good knowledge of anemia? while many of them were previously attended ANC and 40% had nutritional counseling. Please, how you justified this finding?
5. It was not clear the way you measure food security, again you didn’t mention it in the variables
6. How you compare food diversity and food security result in your study? Please check it again??
7. Most of your study participants take meal less than three time per day. again 151 participants were food secured. how you justify it??
8. About 17 women infected with I parasite but only 7 were deworming??
9. Please specified those chronic diseases and the FP utilization time
10. It said “The odds of having anemia among pregnant women who did not take iron-folic acid supplementation were 2.6time.” but no figure show number of px women who did not supplemented IFA?
11. DDS and inter pregnancy interval show big odds ratio with wide CI. do you think it was recommended to have such result?
12. In table 2 the Previous history of ANC follow up was reported by 177 but in table 5 about 226 women had ANC contact indicating that the result was manipulated. similarly check for pregnancy interval. look also it say in the written part 155 women supplemented IFA but no figures a specified in table. At the same time MUAC was not reported in the table of the descriptive. there are many variables not reported in the disruptive tables but show in the table 5
13. The manuscript have no recommendation at all
