# Supplementary material for: The role of iron deficiency and factors associated with anemia during pregnancy in Southeastern Tigray, Ethiopia, 2020
Source: PLoS One. 2025 Feb 10;20(2):e0318275. doi: 10.1371/journal.pone.0318275 (PMC11809803; doi:10.1371/journal.pone.0318275)
Supplement: Attachment — Submitted filename: Response to reviewers.docx [file pone.0318275.s003.docx]

Point by point response to reviewers’ comments

Manuscript number: PONE-D-23-33484

Dear PLOSE ONE Editor,

We are grateful for the opportunity to submit a revised draft of our manuscript titled “Contribution of iron deficiency to anemia and Factors associated with anemia during pregnancy in Southeastern Tigray, Ethiopia, 2020: A cross-sectional study” to PLOSE ONE. We would like to express our gratitude for the time and effort you and the reviewers have dedicated to providing valuable feedback on our manuscript. All the insightful comments and feedbacks from the editor has been corrected accordingly. The insightful comments from the reviewers have also been greatly appreciated, and we have endeavored to incorporate all of the suggestions provided. All changes made to the manuscript have been carefully tracked.

Below, you will find a detailed response to the reviewers’ comments.

| **NO** | **Reviewer comment** | **Response** |
| --- | --- | --- |
|  | **Reviewer 1** | |
| **introduction** | Some of your data in the introduction part was old enough kindly let you replace it with the new information. The claim that contribution of anemia is unknown looks like exaggerated. we know many study conducted in Ethiopia and in Tigray region too. | Dear reviewer, as per your suggestion, we have replaced the outdated information in the introduction with the updated one. Regarding the comment that mentioned the claim about the contribution of anemia being unknown seemed exaggerated, our rationale for that statement was the lack of research in the country assessing both iron deficiency and anemia. However, we have now revised it to "limited studies" instead of "unknown." **(Line 90)** |
| **methods**  A | The study setting for this particular study was not clear? Your study design was facility based cross sectional study however in which particular setting that the study conducted was not noted | Dear reviewer, the study setting for this study was south eastern zone, and four health facilities that are found in south eastern zone were randomly selected to conduct the study among pregnant women who came for ANC follow-up. We have made adjustments to enhance the readability of the text. **(Line 96)** |
| B | Your study population need to be well specific. It stated “pregnant women “ …..it was better if you clearly specified those pregnant women and from where you recruited them? Do you mean pregnant women come for ANC? Or pregnant women come hospital for other purpose? It recommended if you well specified your study population separately. | Dear reviewer, we have modified that accordingly. |
| C | Like that of study population, your study failed to specified the exact inclusion criteria. | We have made the necessary amendment accordingly.**(Line 108-112)** |
| D | Sample size determination you used was from the EDHS 2016 which was population based survey, why you didn’t used the recent study with similar study population | Dear reviewer, we used this large survey because there were limited studies that assessed both iron deficiency and anemia among pregnant women. Moreover, we wanted to get a larger sample size powerful enough to answer our research questions and detect any potential significant associations. |
| E | The calculated sample size was 317. and why not considered the non-response rate. it is common to use include about 10 %or 5% the sample size as non-response rate | Dear reviewer, thank you for your valuable insight. We realized that we failed to explain how we arrived at the final sample size. Here's the explanation: Initially, the calculated sample size was 318. However, due to the lower number of pregnant women with ANC follow-up in the southeastern zone (according to the 2018/2019 report), we applied a correction formula, which resulted in a final sample size of 288. Finally by considering 10% non-response rate the final sample size was 317.**(Line 114-120)** |
| F | The sampling procedure was not clear. Need to specified each. Put number for each steps | Dear reviewer, we have modified and made it specific. We also have put number for each selected health facility accordingly. |
| G | Your study variable is contribution of anemia or anemia??? | Our dependent study variable is anemia. |
| H | The tools for data collection should be cited eg DDS | Dear reviewer, we have cited the references accordingly. **(Line 150)** |
| I | How you check the validity of Hem cue measurement. what was its principle. please specify amount of blood you collected by cuvette | **Dear reviewer,** we verified the validity of the Hem cue measurement by using the precalibrated hem cue measurement and following Standard Operating Procedures (SOPs) and the manufacturer’s instructions for laboratory activities. We always ensure that the analyzer is in the loading position and check for the three flashing dashes, which indicate that the analyzer is ready for use. Additionally, we clean the cuvette holder each day with 70% alcohol and ensure that it is completely dry before re-inserting it.  A drop of capillary blood (10µl) Capillary blood was drawn, through micro cuvettes and inserted into the Hemo Cue Hb analyzer and the result were recorded. **(Line 169)** |
| J | It stated “Finally the punctured part was wiped for any remaining blood from the third drop and covered with an alcohol swab.” Do you think that was simple acceptable you covered that punctured site with alcohol swap?? | Dear reviewer, we were not suggesting that the procedure is just acceptable. Our intention was to demonstrate the blood collection procedures as outlined in the standard operating procedures (SOP) manual. This involves cleaning the fingertip (puncture site) with alcohol and providing a gauze pad to the patient to apply pressure on the puncture site for a minute in order to stop the bleeding. |
| K | How you measure adherence to IFA and the knowledge was not clear | Dear reviewer, The Morisky Medication Adherence Score was used to assess the adherence of pregnant women to IFA supplementation. **(Line 232-233)**  To assess the knowledge of pregnant women about anemia, all the participants were asked ten questions related to anemia, accordingly, the pregnant women were grouped as having good knowledge if they scored greater than or equal to the mean value of correct responses and poor knowledge if they scored less than the mean value of correct responses. (**Line 159-162)** |
| L | What you did for those anemic pregnant women | Dear reviewer, When we encounter pregnant women with anemia, the first step we did was informing them about their anemia status and provide nutritional counseling. We also explain the potential implications of anemia for their health and their baby’s well-being. Following this, we ensure they are linked back to the ANC clinic to receive appropriate healthcare services, including iron folic acid supplementation and further evaluation and management. |
| **Results**  1 | “Number of ANC Visit” the word visit should replaced by contact | We have modified the word accordingly. |
| 2 | In table to of the contraceptive it says “Jagule” I never encounter such named contraceptive kindly specify it | Dear reviewer, It was a typing error and we have modified it as Jadelle. |
| 3 | It recommended that that px women should take meal about four time in day however in your case about three-fourth of participants get food less than three time. Could you please justify it? | Dear reviewer, about three-fourth of participants get food less than or equal to three time, though it is recommended that pregnant women are advised to take a meal about four time a day they typically do not adhere to this recommendation due to the cultural practice of the Tigrian community, as they eat three meals a day (breakfast, lunch, and dinner). |
| 4 | Only about 28% of your participant had good knowledge of anemia? while many of them were previously attended ANC and 40% had nutritional counseling. Please, how you justified this finding? | Dear reviewer, this could be explained due to the fact that nutritional counseling is often general and may not be comprehensive. It mainly focuses on dietary advice, it might not have specifically addressed anemia, plus, the mother’s level of understanding and ability to retain information may also have an impact. Moreover, the ANC care provider may not have enough time to address all nutrition-related issues. |
| 5 | It was not clear the way you measure food security, again you didn’t mention it in the variables | Dear reviewer, as you suggested we have added it in data collection method how we measure food security. **(Line 150-158)**  Dear reviwer, we have mentioned food security in the independent variables list.**(Line 134)** |
| 6 | How you compare food diversity and food security result in your study? Please check it again?? | Dear reviewer, we used a 24-hour recall individual dietary diversity questionnaire for the diet diversity data and, the household food security score was utilized for the food security data. We have rechecked the result, and is correct. |
| 7 | Most of your study participants take meal less than three time per day. again 151 participants were food secured. how you justify it?? | Dear reviewer, this might be explained by the fact that the difference in the time frame for asking the two questions could potentially impact the responses given. Moreover, participants may have enough food but choose to eat fewer meals in order to manage their budget effectively |
| 8 | About 17 women infected with I parasite but only 7 were deworming?? | Dear reviewer, the difference in numbers between parasite infection and deworming may be due to some healthcare provider’s not offering deworming treatment if they do not see the infection as severe. Additionally they might delay deworming until later in pregnancy, following WHO recommendations, due to concerns about the safety of medications for the developing fetus. |
| 9 | Please specified those chronic diseases and the FP utilization time | Dear reviewer, we have specified the time accordingly. The time for FP is before getting pregnant and the time for chronic illness is inclusive, asking for both before and during the current pregnancy. |
| 10**.** | It said “The odds of having anemia among pregnant women who did not take iron-folic acid supplementation were 2.6time.” but no figure show number of px women who did not supplemented IFA? | Dear reviewer, Thank you for your valuable insight, we realized that we neglected to include the variable for IFA intake during the current pregnancy, which was originally responded to as either 'Yes' or 'No'. We have since rectified this and included the variable in the results. **(Table 3 row 6)** |
| 11 | DDS and inter pregnancy interval show big odds ratio with wide CI. do you think it was recommended to have such result? | Dear reviewer, we have rerun the analysis to ensure the findings and The results for both DDS and interpregnancy gap remain consistent .The odds ratio was also supported by other studies conducted in the country. |
| 12 | In table 2 the Previous history of ANC follow up was reported by 177 but in table 5 about 226 women had ANC contact indicating that the result was manipulated. similarly check for pregnancy interval. look also it say in the written part 155 women supplemented IFA but no figures a specified in table. At the same time MUAC was not reported in the table of the descriptive. there are many variables not reported in the disruptive tables but show in the table 5 | Dear reviewer, thank you for identifying these discrepancies in the tables and pointing out the inconsistencies between the reported numbers. We have addressed these discrepancies by carefully reanalyzing the data. Upon review, we realized that we had omitted some important variables from the result tables. Specifically, we failed to include IFA supplementation, gestation age, and nutritional status (MUAC) in the initial results table. These variables have now been added to the results table. Additionally, we acknowledge the discrepancy in the number of ANC. After reanalyzing the data, we confirmed that a typing error had occurred during the insertion of the number in the results section. This error has been rectified accordingly. (**Table 2 row 3, Table 3 row 6 and 8)** |
| 13 | The manuscript have no recommendation at all | Dear reviewer, Thank you for your valuable insights. The recommendation part was unintentionally omitted but we have rectified this by incorporating the recommendation along with the conclusion. **(Line 457-463)** |
|  | **Reviewer** **2** | |
| 1 | General: The manuscript has some grammatical errors that need to be fixed. For example, lines 70 and 499. It also needs formal writing, which must be different from a thesis. | Dear reviewer, Thank you for pointing out the grammatical errors in the manuscript. Following a comprehensive review, we have diligently rectified the inconsistencies and refined the language to ensure its utmost clarity. Moreover, we have refined the manuscript to a more formal writing style to align with the requisite standards of the journal. |
| 2 | Some polishing is needed on the grammar. Integrating lines 59–62 with other sections would be preferable | Dear reviewer, we have thoroughly addressed the grammatical errors and made an effort to integrate lines 59–62 with other sections, in accordance with your recommendations. |
| 3 | The study design and setting should be combined with the study area section. Words like "first cuvette"- is there a second cuvette for one subject? "third drop," etc. are inappropriate in this context, so lines 159–166 must be rewritten. | Dear reviewer, we have made the necessary modifications and rewritten the paragraph accordingly. |
| 4 | A brief, succinct, and formal writing style is required in the data quality assurance section | Dear reviewer, we have made the necessary amendments for the data quality assurance section. **(Line 192-205)** |
| 5 | There are a few grammar mistakes that should be fixed. Consider line 499. | Dear reviewer, thank you for your insight and we have made the necessary corrections for grammatical and punctuation errors accordingly. |

We think we have addressed the points you have raised and we look forward to hearing from you in due time regarding our revised manuscript and to respond to any further questions and comments you may have.

Sincerely,

Tedros Bereket (Corresponding author)

Email: Tedros.berekt99@gmail.com

.
