# Supplementary material for: The role of iron deficiency and factors associated with anemia during pregnancy in Southeastern Tigray, Ethiopia, 2020
Source: PLoS One. 2025 Feb 10;20(2):e0318275. doi: 10.1371/journal.pone.0318275 (PMC11809803; doi:10.1371/journal.pone.0318275)
Supplement: Attachment — Submitted filename: Response to editor & reviewers.docx [file pone.0318275.s004.docx]

Point by point response to Editor and reviewers’ comments

Manuscript number: PONE-D-23-33484R1

Dear PLOSE ONE Editor,

We are grateful for the opportunity to submit a revised draft of our manuscript titled “The role of iron deficiency and Factors associated with anemia during pregnancy in Southeastern Tigray, Ethiopia, 2020: A cross-sectional study” to PLOSE ONE. We would like to express our gratitude for the time and effort you and the reviewers have dedicated to providing valuable feedback on our manuscript. All the insightful comments and feedbacks from the editor has been corrected accordingly. The insightful comments from the reviewers have also been greatly appreciated, and we have endeavored to incorporate all of the suggestions provided. All changes made to the manuscript have been carefully tracked.

We have not cited papers that have been retracted, and we have modified to our reference citation as per the comments.

Below, you will find a detailed response to the Editor previous and current comments as well as reviewers’ comments.

| **NO** | **Previous and current editors comment** | **Response** |
| --- | --- | --- |
| 1 | Line 25: Therefore, this study was aimed at assessing the contribution……..’ It is better written as, ‘Therefore, this study aimed to assess the contribution……… | Dear editor, we have made the correction based on your suggestion. Line 24-36 |
| 2 | Line 28-30: ‘…….was conducted on 311 pregnant women who were attending four health facilities in the southeastern zone of Tigray region between January 2020 and June 2020.’ It is better written as, ‘………was conducted among 311 pregnant women who attended four health facilities in the southeastern zone of Tigray region from January 2020 to June 2020.’ | Dear editor, we have made the necessary corrections in accordance with your feedback. Line 27-29 |
| 3 | What about ‘Bivariate logistic regression’ and ‘Multivariate logistic regression’ in the abstract? | Dear editor, we have made the necessary adjustments accordingly. Line 30-32 |
| 4 | In the abstract, the p-value should be included. | Dear editor, we have made the correction based on your suggestion and added the P value in the abstract. line 31-32 |
| 5 | In the abstract and result, the absolute number (numerator and denominator) is needed together with the percentage. For example, A/B (17.7%). | Dear Editor, we have promptly made the necessary corrections and included the numerator alongside the denominator, along with the percentage |
| 6 | Line 30: ……..to collect data.’ It is better written as, ‘………to collect the data’ | Dear Editor, we have revised the text accordingly. |
| 7 | The introduction requires revision. The rationale is not explained well. Why you did this research? What has been done before, what is known before and which you intend to fill? | Dear Editor, we have revised the introduction and made the necessary adjustments accordingly. |
| 8 | Line 100: Rewrite as, ‘Materials and Methods’ | Dear Editor, we have corrected and rewrite it as Material and Methods. |
| 9 | Line 102: The study was conducted among 311 pregnant women from January 2020 to June 2020…. | Dear Editor, we have modified the statement as per your comment. |
| 10 | Line 112: Rewrite as, ‘Study design’, because the ‘setting’ is already described in the study area, period and population. | Dear Editor, we have made the necessary amendment accordingly. |
| 11 | Line 140: Rewrite as, Data collection | Dear Editor, we have corrected it as Data collection. |
| 12 | Line 158: Better be, ‘Laboratory Methods’ | Dear Editor, we have corrected it as laboratory methods. |
| 13 | The ‘data quality assurance’ portion should be rewritten. Select the most important and present. | Dear Editor, we have rewrite the data quality assurance portion and selected the most important only. |
| 14 | Revision was need in the’ data analysis’ section. Binary? Do you want to say ‘Bivariate logistic regression’ and ‘Multivariate logistic regression?’ Additionally, the statement ‘First, descriptive statistics of the study variables were done and data were presented using tables, and figures’ should be removed | Dear Editor, we have made the necessary amendments as per your comment and removed ‘First, descriptive statistics of the study variables were done and data were presented using tables, and figures’ from the data analysis section. |
| 15 | A separate heading ‘Conclusion’ is required. If there is any recommendation you can add here. | Dear Editor, we have made a separate heading conclusion and add recommendation in that section. |
| 16 | Study variables should be written as ‘Dependent variable’ and ‘Independent variables’ clearly and separately. No description will be needed here, for example, 0 and 1. | Dear Editor, we have corrected it accordingly and write the dependent and independent variable separately. |
| 17 | Line 252: Socio-demographic characteristics of study participants | Dear Editor, we have corrected it as per your comment. |
| 18 | Why you leave P-value in the ‘associated factors’ assessment ‘table’? | Dear Editor, I didn’t leave the P-value; I represented it with single and double asterisks. I also wrote the reference marks for the symbols in the footnote below the table. |
| 19 | Write the ‘operational definitions’ to the standard | Dear Editor, we have modified the operational definition to the standard accordingly. |
| 20 | Put the description of supplementary file (s) below the reference lists | Dear Editor, we have put the description of supplementary file below the reference as per your comment. |
| 21 | Keeping appropriate statements selected in the submission system, you can remove the following from the main manuscript.  Consent for publication  Availability of data and materials  Competing interest | Dear Editor, we have removed those from the manuscript and write it in the submission system appropriately. |
| 22 | - The references were not written properly. All references should be written correctly according to Vancouver style. - Use the alignment ‘Justify’ for the text throughout the manuscript - Follow the standard binomial nomenclature, italize journal name and the word ‘et al’ | Dear Editor, we have updated the references accordingly and applied the 'Justify' alignment for the text throughout the manuscript, ensuring we followed the standard binomial nomenclature. |

We think we have addressed the points you have raised and we look forward to hearing from you in due time regarding our revised manuscript and to respond to any further questions and comments you may have.

Sincerely,

Tedros Bereket (Corresponding author)

Email: Tedros.berekt99@gmail.com

.
